# Supplementary material for: Epigenomic landscape of the developing human rhombic lip reveals gene regulatory network and non-coding loci of developmental, evolutionary, and disease relevance
Source: bioRxiv. 2025 Oct 30:2025.10.30.685586. Preprint. [Version 1] doi: 10.1101/2025.10.30.685586 (PMC12636629; doi:10.1101/2025.10.30.685586)
Supplement: Supplement 1 [file NIHPP2025.10.30.685586v1-supplement-1.pdf]

bioRxiv preprint doi: https://doi.org/10.1101/2025.10.30.685586; this version posted October 31, 2025. The copyright holder for this preprint (which was not certified by peer review) is the author/funder, who has granted bioRxiv a license to display the preprint in perpetuity. It is made available under aCC-BY 4.0 International license.

Supplementary Figures

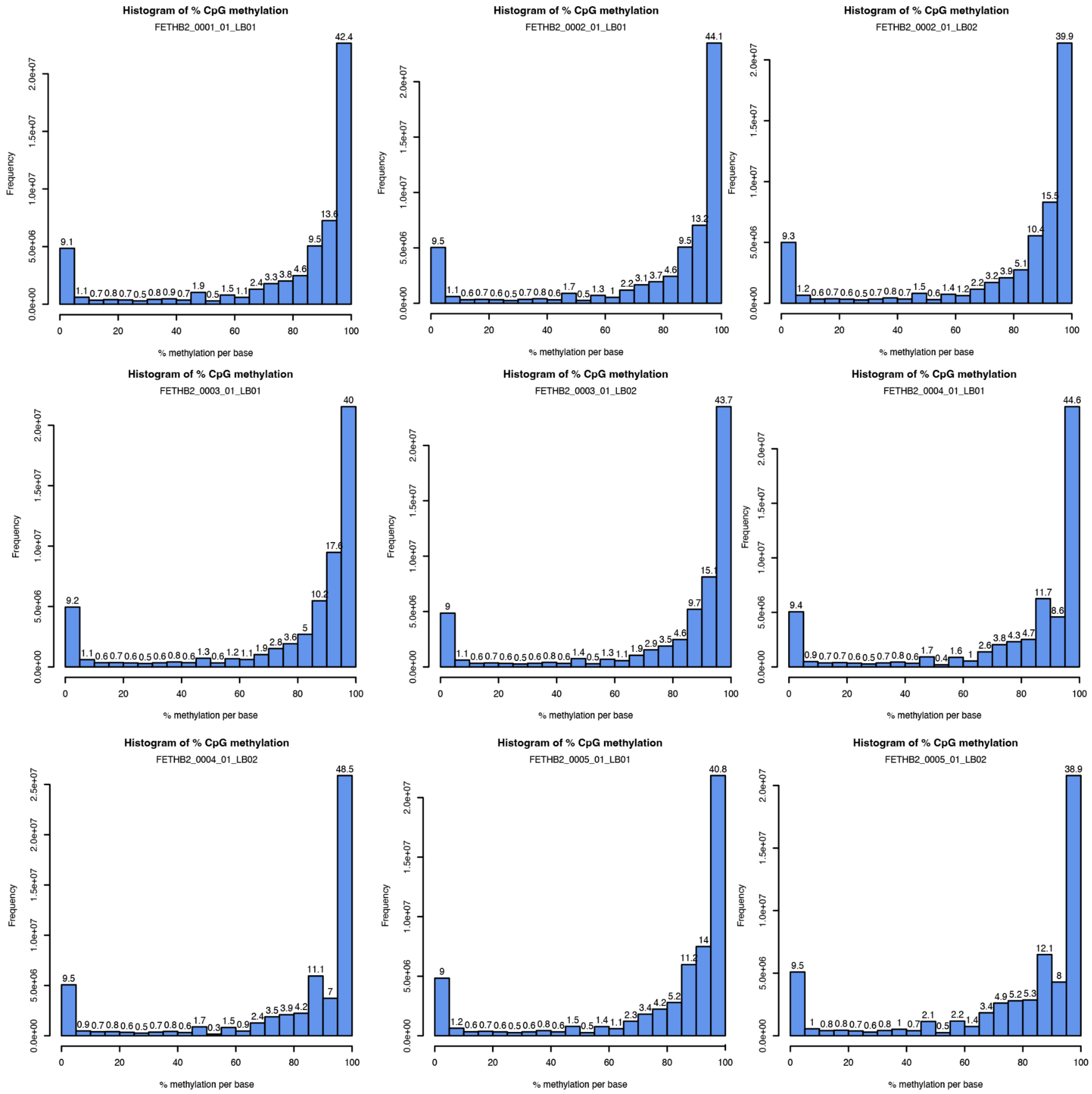

Supplementary Figure 1. Genome-wide distribution of base-level percent DNA methylation. Each panel shows data for an individual DNA methylome.

bioRxiv preprint doi: https://doi.org/10.1101/2025.10.30.685586; this version posted October 31, 2025. The copyright holder for this preprint (which was not certified by peer review) is the author/funder, who has granted bioRxiv a license to display the preprint in perpetuity. It is made available under aCC-BY 4.0 International license.

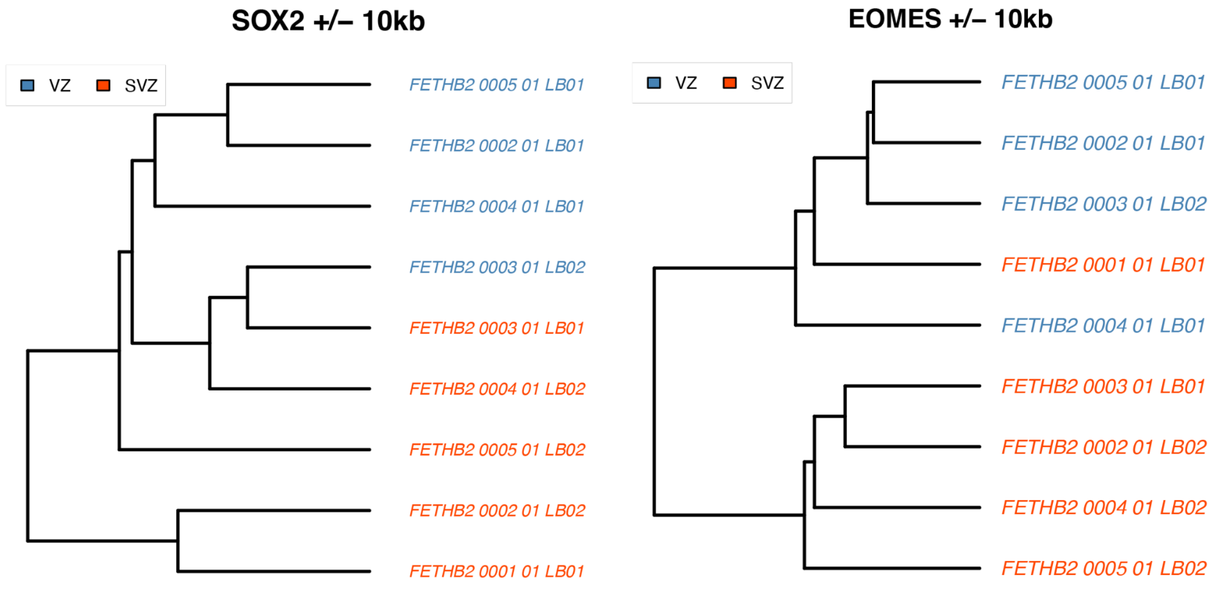

Supplementary Figure 2. Hierarchical clustering of DNA methylomes based on CpG methylation of (a) the SOX2 gene region (b) EOMES gene region.

bioRxiv preprint doi: https://doi.org/10.1101/2025.10.30.685586; this version posted October 31, 2025. The copyright holder for this preprint (which was not certified by peer review) is the author/funder, who has granted bioRxiv a license to display the preprint in perpetuity. It is made available under aCC-BY 4.0 International license.

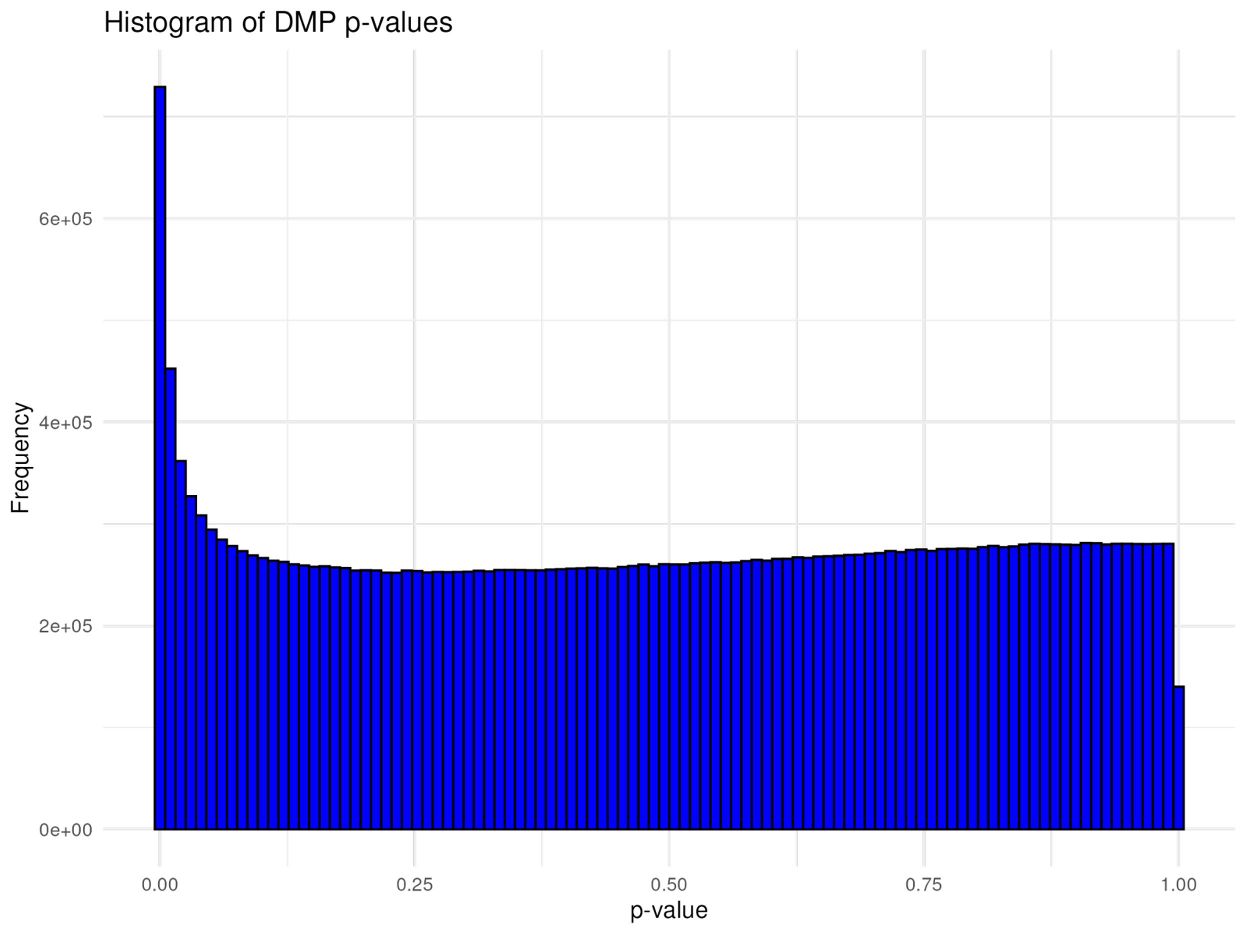

Supplementary Figure 3. Distribution of nominal p-values of base-level differentially methylated CpGs between rhombic lip ventricular zone and subventricular zone.

bioRxiv preprint doi: https://doi.org/10.1101/2025.10.30.685586; this version posted October 31, 2025. The copyright holder for this preprint (which was not certified by peer review) is the author/funder, who has granted bioRxiv a license to display the preprint in perpetuity. It is made available under aCC-BY 4.0 International license.

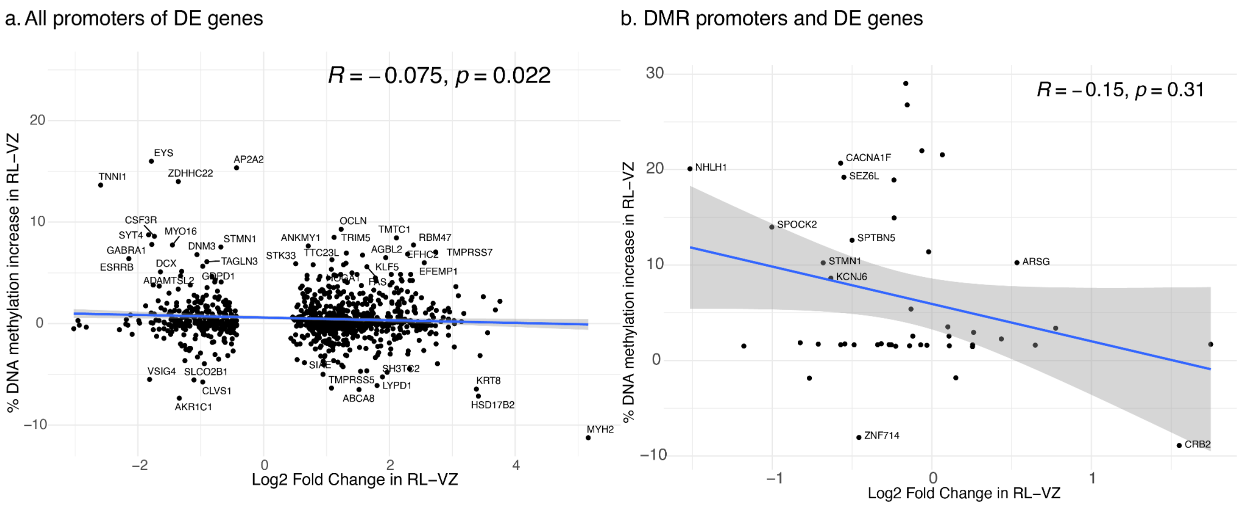

Supplementary Figure 4. Promoter-level DNA methylation and gene expression.

a. Promoter-level DNA methylation increase and transcription increase of the corresponding gene; analysis limited to significantly differentially expressed genes (N=961 genes). Spearman correlation and corresponding p-value from a t-test are shown.

b. Promoter-level DNA methylation increase and transcription increase are limited to genes where the promoter region overlaps a DMR and which is differentially expressed in the rhombic lip (N=45 genes). Spearman correlation and corresponding p-value from a t-test are shown.
